# An Overview of Nano Multilayers as Model Systems for Developing Nanoscale Microstructures

**DOI:** 10.3390/ma15010382

**Published:** 2022-01-05

**Authors:** Chelsea D. Appleget, Juan Sebastian Riano, Andrea M. Hodge

**Affiliations:** 1 Department of Aerospace and Mechanical Engineering, University of Southern California, Los Angeles, CA 90089, USA; capplege@usc.edu; 2 Mork Family Department of Chemical Engineering and Materials Science, University of Southern California, Los Angeles, CA 90089, USA; rianozam@usc.edu

**Keywords:** magnetron sputtering, nanometallic multilayers, interfaces, grain boundary energy, coherency, binary alloys, nanomaterials

## Abstract

The microstructural transformations of binary nanometallic multilayers (NMMs) to equiaxed nanostructured materials were explored by characterizing a variety of nanoscale multilayer films. Four material systems of multilayer films, Hf-Ti, Ta-Hf, W-Cr, and Mo-Au, were synthesized by magnetron sputtering, heat treated at 1000 °C, and subsequently characterized by transmission electron microscopy. Binary systems were selected based on thermodynamic models predicting stable nanograin formation with similar global compositions around 20–30 at.%. All NMMs maintained nanocrystalline grain sizes after evolution into an equiaxed structure, where the systems with highly mobile incoherent interfaces or higher energy interfaces showed a more significant increase in grain size. Furthermore, varying segregation behaviors were observed, including grain boundary (GB) segregation, precipitation, and intermetallic formation depending on the material system selected. The pathway to tailored microstructures was found to be governed by key mechanisms and factors as determined by a film’s initial characteristics, including global and local composition, interface energy, layer structure, and material selection. This work presents a global evaluation of NMM systems and demonstrates their utility as foundation materials to promote tailored nanomaterials.

## 1. Introduction

Nano-metallic multilayers (NMMs) provide an unparalleled nanostructured working space due to their periodic stacking structure that enables control of the layer thickness, the local composition and the density and structure of the interfaces [1,2]. As such, NMMs allow for microstructural features that can be tailored while providing limitless compositional space. These characteristics provide a foundational scientific testbed to study and surmount limitations encountered in nanostructured materials including lack of thermal stability and ductility, as well as being limited to finite material systems [3,4,5,6,7].

NMMs have been achieved through methods such as accumulative roll bonding [8,9], chemical vapor deposition [10,11], electrodeposition [12,13] and physical vapor deposition (PVD) [14,15]. PVD techniques, specifically magnetron sputtering, expand the possible material systems that may be synthesized as NMM configurations while promoting control over initial features such as interfaces and grain structure. Therefore, NMMs can leverage the favorable characteristics of nanostructured materials that can be credited to the high density of grain boundaries (GBs) and interfaces while expanding possible microstructural arrangements. As a result of these valuable attributes, NMMs have demonstrated capability as model systems for developing new equiaxed nanostructured materials [16,17,18,19,20,21]. Individual studies on different resulting microstructures are highly encouraging [22,23,24], but what remains in question is an overarching view of the mechanistic relationships between different initial multilayer structures and the final nanostructure achieved after thermally-driven microstructural transformations [25].

In general, thermal studies of NMMs have traditionally focused on preserving the integrity of the layers rather than using NMMs as a route towards achieving equiaxed nanostructured materials [26,27,28]. However, by using stability maps of binary systems for identifying promising alloy compositions [29,30,31], sputtered multilayered configurations can be selected as a route to promote equiaxed nanocrystalline structures [16]. These maps, as determined by thermodynamic [32,33,34] models, can be combined with the highly tunable NMMs to allow for the study of shifting processes and mechanisms during microstructural formation at the nanoscale.

As such, this manuscript provides an overarching evaluation of thoroughly characterized systems to isolate the key factors and mechanisms that are driven by the initial NMM characteristics. The contributions of both interface properties and composition are generalized to identify global relationships between predictions of nanoscale microstructures and the final observed nanostructured microstructures. In this article, we present the microstructural synergies and differences within four distinct model systems (Hf-Ti [18], Ta-Hf [19], W-Cr [20], Mo-Au [17]) in their as-sputtered condition and after annealing at 1000 °C for 96 h. These model binary material systems were chosen because they are all predicted to exhibit nanocrystalline stability at high temperatures [30,31]. Furthermore, these systems are non-equiatomic to promote enhanced diffusion on the path toward multi-layered-to-equiaxed microstructural transformations. This approach assesses the relationship between initial characteristics and heat-treated microstructures as a pathway to develop highly tailored nanoscale microstructures.

## 2. Materials and Methods

Hf-Ti, Mo-Au, Ta-Hf and W-Cr NMMs (2 µm total thickness), comprising alternating mixed and pure solute layers, were deposited on Si (100) substrates via DC magnetron sputtering following procedures described in previous manuscripts [17,18,19,20]. In these manuscripts, more detailed discussions of both the experimental approach and findings can be found. These binary systems were selected because they are predicted via thermal stability maps to retain nanograin structures under annealing. The global composition of the multilayers was targeted to be around 20 at.% solute concentration for a non-equiatomic solute:solvent ratio, and was achieved by adjusting the sputtering rates, whereas the individual layer thicknesses were controlled by tuning the sputtering time. The properties of these as-sputtered NMM films are summarized in Table 1. After deposition, surface profiles of the films were collected using an AMBIOS profilometer (KLA, Milpitas, CA, USA) to calculate residual stresses, while their crystallographic texture was studied through XRD scans collected using a Rigaku Ultima IV diffractometer (Rigaku, Tokyo, Japan). The films were subsequently removed from the substrates by a short annealing at 800 °C (1 min holding, 10°/min ramping), where these short processes were confirmed to not alter the as-sputtered microstructures. The thicknesses and compositions of the multilayers were examined using a JSM-7001 scanning electron microscope (JEOL, Tokyo, Japan) fitted with an energy-dispersive X-ray spectroscopy (EDS) EDAX detector. Differential scanning calorimetry (DSC) scans of the films were collected in a Labsys thermal analyzer (KEP Technologies, Mougins, France) from 20 to 1000 °C, heating the multilayers at a rate of 10 °C/min under constant flow of 40 mL Ar/min, to explore the microstructural evolution of each system leading to the formation of nanostructures. For these scans, the enthalpy of the endothermic and exothermic transformations was measured over a baseline that was extracted using Origin Lab. In order to experimentally observe the microstructural changes correlating to these transformations, each multilayer system was annealed for 96 h at various temperatures up to 1000 °C inside a GSL1100X vertical tube furnace (MTI Corporation, Richmond, CA) under vacuum (5 × 10^−4^ Pa) [17,18,19,20]. At the end of the heat-treatment, the samples were rapidly quenched in a low vapor pressure oil. The initial as-sputtered and final tailored microstructures (1000 °C) are the focus of this article in order to capture a more general understanding of the concluding microstructural transformations where details on intermediate microstructures can be found elsewhere [17,18,19,20]. Transmission electron microscopy (TEM) lamellas exposing the cross-section of the films were prepared via focus ion beam (FIB) lift-out techniques within a JEOL FIB-4500 microscope (JEOL, Tokyo, Japan), where the surface was protected from beam damage with a thin layer of C. The microstructure and local compositions of the films were studied via TEM imaging of the lamellas in a JEOL JEM-2100F TEM (JEOL, Tokyo, Japan) equipped with an Oxford X-MaxN 100 EDS detector. Multiple TEM dark field micrographs were collected to measure the projected areas of grains, which were later used to calculate grain size distributions following procedures described in previous works [17,18,19,20]. Several diffraction techniques such as selected area electron diffraction (SAED) and nanodiffraction were used to determine grain orientations.

## 3. Results and Discussion

In multilayered structures, key features such as characteristic layer thickness, coherency, lattice mismatch, initial grain size, and material selection all contribute to the initial microstructure, which in turn dictate the microstructural evolution under heat treatment. By evaluating the microstructures before and after thermal transformations across discrete material systems (Hf-Ti, Ta-Hf, W-Cr, Mo-Au), the effect of fundamental controlling parameters can be compared. Furthermore, heat-treatment temperatures of 1000 °C are above the benchmark for typical grain growth behavior studies in nanocrystalline materials [35,36,37] and is where most transitions to a fully equiaxed microstructure are expected across the various binary alloys.

**Figure 1:**
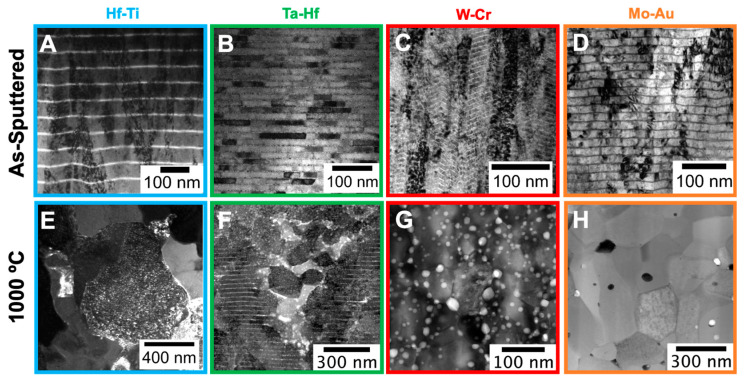
Cross-sectional transmission electron microscopy (TEM) images of the as-deposited multilayer films (**A**–**D**) and nanostructured films after heat treatment at 1000 °C (**E**–**H**). The micrographs feature the four nanometallic multilayer systems: Hf-Ti (**A**,**E**), Ta-Hf (**B**,**F**), W-Cr (**C**,**G**), and Mo-Au (**D**,**H**) [17,18,19,20].

Overviews of these NMM-to-nanocrystalline microstructures are presented in Figure 1, where the as-sputtered (ASP) and post heat-treatment (96 h at 1000 °C) microstructures of the four systems are shown in cross-sectional TEM images. In order to compare initial and final microstructural details, these micrographs are in bright field (BF) mode and, therefore, show mass contrast. Distinct layers are visible in each system before heat-treatment (Figure 1A–D). The as-sputtered Hf-Ti and W-Cr show columnar grains that continue through multilayer interfaces, while the as-deposited Ta-Hf and Mo-Au comprised non-continuous grains throughout the stratified structure. As for post-heat treatment at 1000 °C, equiaxed grains are observed across all binary alloys (Figure 1E–H), with some notable differences in the NMM transformations between systems. The W-Cr and Mo-Au systems both indicate full development into an equiaxed morphology, with precipitates forming both at the GBs (Figure 1G) and within the grains (Figure 1H). Figure 1E shows that at 1000 °C, Hf-Ti is also equiaxed, but with much larger Hf-rich grains surrounded by crystallized, impinged Ti grains. In Ta-Hf (Figure 1F), there is a combination of columnar grains with well-defined layers (outside edges) as well as equiaxed grains with Hf-rich GB precipitates and clustering (center). Overall, these heat-treated NMMs show drastic microstructural transformations with limited grain growth, but the final microstructures differ in key aspects depending on the alloy system, thus indicating that multiple mechanisms are present in the route to equiaxed microstructures.

**Figure 2:**
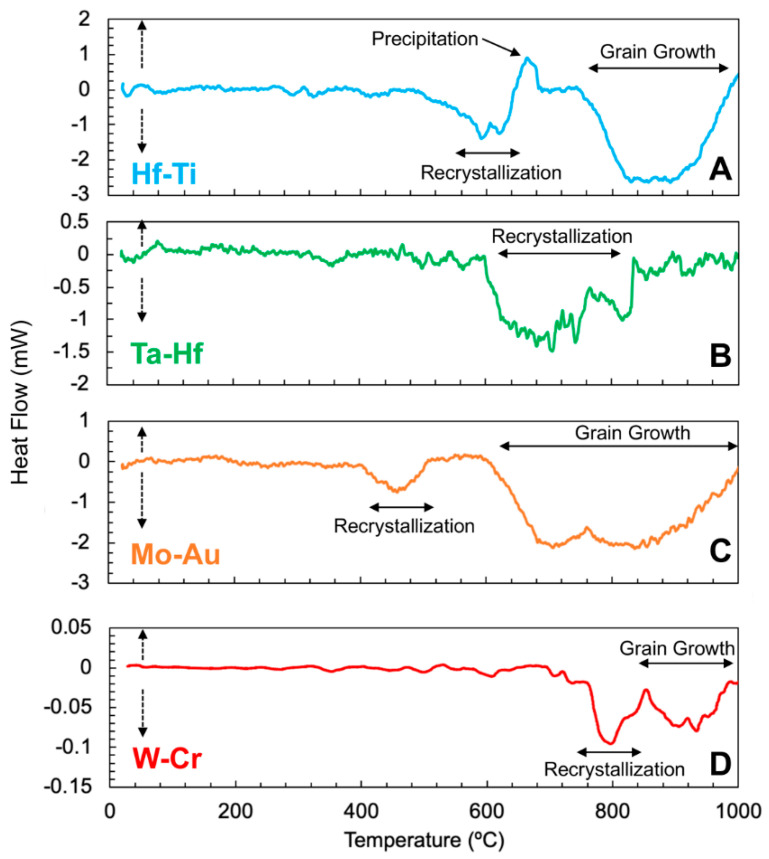
Differential scanning calorimetry (DSC) scans of the nanometallic multilayers from room temperature to 1000 °C showing multiple transitions for each of the systems: (**A**) Hf-Ti, (**B**) Ta-Hf, (**C**) Mo-Au, and (**D**) W-Cr. Endothermic reactions are indicated by the dashed positive vertical arrow and exothermic reactions are indicated by the dashed negative vertical arrow [17,18,19,20].

To compare and contrast amongst the NMM systems and explore the competing mechanisms and processes, the DSC scans of the NMM systems (Figure 2) highlight various critical temperature-induced events, including the stages of recrystallization, precipitation, and grain growth expected in each system. Coupled with the cross-sectional micrographs (Figure 1), the mechanisms controlling the evolution of the heat-treated NMMs can be examined. These scans suggest that at 1000 °C, Hf-Ti, Mo-Au, and W-Cr will have all undergone recrystallization and grain growth, while Ta-Hf will have only undergone recrystallization. This is in agreement with the TEM cross-sections at 1000 °C, where Ta-Hf (Figure 1F) has retained some of the initial multilayered structure, while the Hf-Ti, Mo-Au, and W-Cr systems are fully transformed, now comprised of equiaxed grains surrounded by precipitates at the grain boundaries. Furthermore, these DSC results suggest that at the temperatures required for recrystallization and grain growth, the diffusivities are large enough in magnitude that higher grain boundary mobility can yield diffusion zone formation that initiates new phases [20,38,39]. In the Hf-Ti NMMs, the Ti has segregated to the grain boundaries and induced clusters, which then formed a wetting complexion that thermodynamically stabilized the recrystallized grains, resulting in the formation of a heterogenous microstructure [18]. In both Mo-Au and W-Cr, precipitation (Figure 1H) and intermetallic formation (Figure 1G) are observed along the GBs, at triple junctions, and within grain interiors have been observed experimentally.

**Figure 3:**
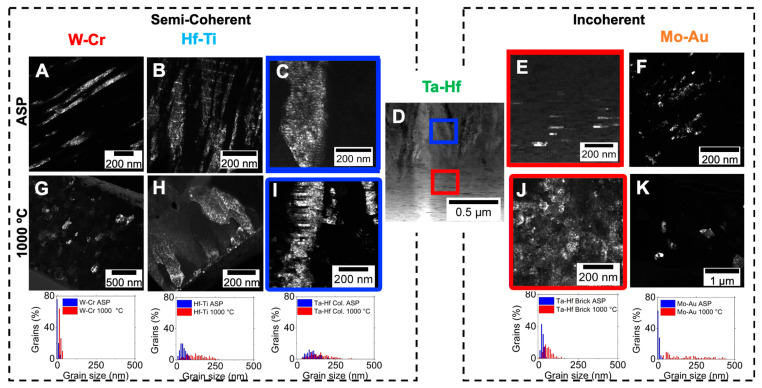
Overview of microstructural evolution of semicoherent (left) and incoherent (right) Nano-metallic multilayers (NMMs). Dark field (DF) TEM and corresponding grain size distributions for as-deposited multilayers (**A**–**F**) and annealed at 1000 °C (**G**–**K**). Please note that the microstructure shown in the middle, Ta-Hf (**D**), has a bimodal microstructure, with columnar semicoherent grains, noted in blue (**C**,**I**), and brick-like incoherent grains, highlighted in red (**E**,**J**). Corresponding grain size distribution for as deposited (blue) and annealed (red) samples are shown below for the respective multilayers.

Further microstructural analysis can be included to identify key features such as grain size and shape as shown in Figure 3A–K, where dark field (DF) TEM images of the NMM systems before and after heat-treatment at 1000 °C and corresponding grain size histograms are presented. The NMM systems are organized by the coherency of the as sputtered binary alloys, semicoherent on the left (W-Cr, Hf-Ti) and incoherent on the right (Mo-Au). In general, heterophase interfaces, or interfaces separating two crystals of different compositions, can be divided into three classes: coherent, semicoherent, and incoherent. Fully coherent interfaces exhibit long-range order, whereas incoherent interfaces exhibit no long-range order [40]. Semicoherent interfaces are characterized by crystallographic mismatch that can be accommodated by periodic misfit dislocations at the interface. Lattice mismatch, or the degree of atomic matching between two adjacent crystals, is another way of quantifying heterostructure interfaces. Coherent and semicoherent interfaces, those with little to no lattice mismatch, can form subgrains of uninterrupted columnar structures over heterophase interfaces, whereas incoherent interfaces can be highly mobile and interrupt the columnar grain structure [41,42,43,44]. Ta-Hf is a special case for coherency of interfaces, where a bimodal microstructure with two different regions is present: columnar and brick-like grains. The Ta-Hf columnar grains (blue) are semicoherent and the brick-like grains (red) are incoherent. 

In the as-sputtered semicoherent systems (Figure 3A–C: W-Cr, Hf-Ti, columnar Ta-Hf), grains span the layer interfaces. This is in contrast to the as-sputtered incoherent systems (Figure 3D–F: brick-like Ta-Hf, Mo-Au) where grains are restricted and terminate at the layer interfaces, therefore the as-sputtered layer thickness controls the initial grain size. At 1000 °C (Figure 3G–K) the DF TEM cross-sections indicate that all NMMs have transformed considerably from their as-sputtered microstructures, while maintaining nanoscale grain structure. With the exception of columnar Ta-Hf (Figure 3I), all systems exhibit equiaxed microstructures comprised of nanocrystalline grains after heat treatment. The grain size differences in the as-sputtered and heat-treated samples were quantified by calculating the grain size distributions. For the semicoherent systems, the histograms illustrate the narrower grain distribution before and after heat treatment for W-Cr (8 ± 5 to 78 ± 32 nm), followed by Hf-Ti (48 ± 19 to 132 ± 62 nm) and the columnar Ta-Hf (109 ± 49 to 157 ± 73 nm). For the incoherent interfaces, the brick-like Ta-Hf (31 ± 9 to 60 ± 28 nm) showed a narrow distribution while Mo-Au (9 ± 5 to 190 ± 121 nm) exhibited a wide dispersion in grain size. Here the bimodal NMM structure of Ta-Hf enables an unprecedented link between microstructural transformations and interface structure by existing in two coherency regimes. Although all systems maintained nanocrystalline grain sizes after thermal transformation, the systems with incoherent interfaces showed a more significant increase in grain size after heat treatment. This is expected, as coherent interfaces have lower energy due to the higher degree of bonding, while incoherent interfaces are at a higher energy state and are therefore expected to be more mobile. Some key differences in the microstructural transformations of these alloy systems can be attributed to their interface properties, but coherency does not encompass all mechanisms at play.

**Figure 4:**
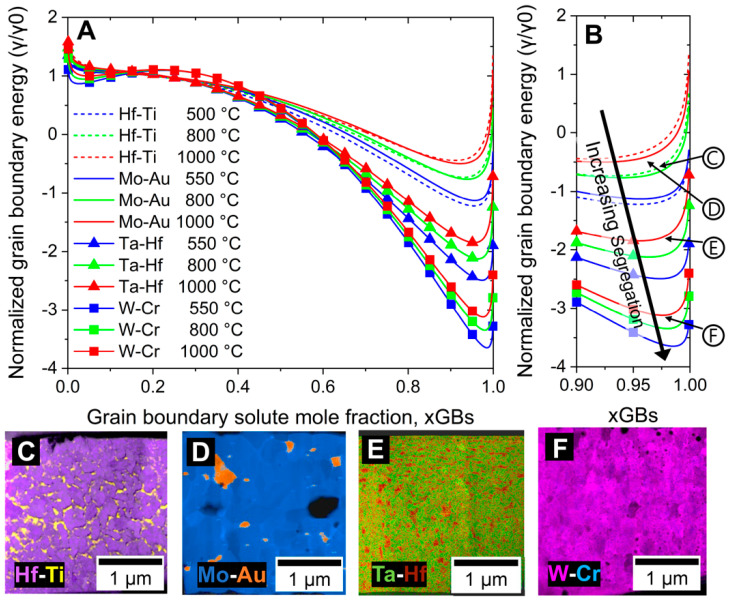
(**A**) Normalized grain boundary energy calculations as a function of grain boundary solute mole fraction at annealing temperatures ranging from 500 to 1000 °C. The inset in (**B**) shows a zoomed view of the normalized grain boundary energy minima where the segregation to the GBs is energetically favorable. In (B) circled labels (**C**–**F**) correspond to EDS snapshots which indicate varying levels of GB segregation for (**C**) Hf-Ti, (**D**) Mo-Au, (**E**) Ta-Hf, and (**F**) W-Cr.

There is a wide range of factors that can influence the final tailored microstructure in addition to coherency; for example, as films undergo heat treatment, the local composition will be changing, thus modifying the grain boundary energy (GBE) as a function of the elemental distributions. As the microstructure evolves, the local alloying content of the binary NMM will change as the global composition stays the same. Furthermore, the high density of interfaces in a NMM structure can shift the kinetics of segregation in alloys by decreasing the diffusion length scales. As observed in Figure 1 and Figure 3, the final microstructures exhibit different types of segregation not directly related to their coherency (or lack thereof). Figure 4 presents how GBE changes due to compositional variations, while highlighting critical segregation points for each system. Composition is known to be important in governing segregation behavior, where segregation continues until the GB is close to compositional saturation and the grain boundary energy is minimized [40]. To illustrate this relationship, the normalized grain boundary energy, with respect to the solvent, was computed as a function of the molar solute content at the grain boundary at 500, 800, and 1000 °C using the Darling et al. modification of the Wynblatt and Ku model, in which the chemical and elastic enthalpies of segregation are estimated using the Defay and the McLean equations, while the entropy of segregation is that of an ideal regular solution [34,45,46]. The evaluation of these terms, as described in previous publications, only requires the binary mixing enthalpies, which were obtained from the tables constructed by Murdoch and Schuh using Miedema’s model, and several mechanical constants commonly reported in the literature [18,30,43]. The results of these calculations, presented in Figure 4A,B, indicate that the grain boundary energy follows the progression of Hf-Ti (ΔH_mix_ ≈ 14.47 kJ/mol) > Mo-Au (ΔH_mix_ ≈ 16.36 kJ/mol) > Ta-Hf (ΔH_mix_ ≈ 23.73 kJ/mol) > W-Cr (ΔH_mix_ ≈ 38.68 kJ/mol), indicating a strong dependence on the energy of mixing. In Figure 4A, at higher temperatures for a given system, the GBE minima shift to the left, meaning that a lower amount of solute is needed to minimize the GBE. Although this is a simplified view of grain boundary solute segregation behavior [47,48,49], it does support the assertion that at elevated temperatures with high solute concentrations, GB precipitates can nucleate and grow until reaching a GBE-stabilized state. This is because precipitates can form at enriched grain boundaries because of the formation of diffusion zones, which are areas near the GB that have a compositional gradient. At these locations there will be areas where the compositions are such that after atomic rearrangement, a new phase with lower associated Gibbs energy will form, this new phase is the precipitate. Further details on the increasing segregation patterns can be observed in the magnified region in Figure 4B.

To complement the GBE calculations and TEM cross-sections (Figure 1A–H and Figure 4), the Figure 4 insets C-F illustrate EDS maps at critical points in the microstructural transformations. In semicoherent Hf-Ti heat treated to 800 °C (Figure 4C, Ti (yellow) segregation has reached grain boundary saturation around 91 at.%Ti after recrystallization. The saturation of Ti (yellow) at the grain boundaries lowers the GBE. After GB saturation is achieved, cluster formation and further Ti precipitation occurs, with precipitates surrounding the Hf-rich grains (purple). Upon further heat treatment, Ti precipitates crystallize and grain growth ensues (Figure 1E). The EDS map for Mo-Au (Figure 4D) indicates phase separation of Mo (blue) grains with Au (gold) precipitates. At 1000 °C, Au precipitates are visible along the Mo GBs, at triple junctions, and within the grains. Although a similar segregation is expected for both Hf-Ti and Mo-Au systems, the microstructural progression was accelerated in Mo-Au due to the presence of incoherent interphases that enhanced grain boundary diffusion. The Ta-Hf (Figure 4E) system exhibits Ta-rich (green) grains surrounded by Hf-rich (maroon) precipitates at the GBs. The Ta precipitates form a wetting complexion that reduces the energy and, therefore, mobility of the GBs, thus stabilizing the newly equiaxed grains. At 1000 °C, W-Cr (Figure 4F contains W-rich (magenta) equiaxed grains surrounded by amorphous W_3_Cr precipitates. The presence of W_3_Cr precipitates was indicated by high-resolution TEM (HRTEM) EDS measurements and corresponding selected area electron diffraction (SAED) patterns as outlined in [20]. The localization of the W_3_Cr intermetallic precipitates to the GBs suggest that during precipitation there are nanocrystalline amorphous Cr clusters (blue) at the grain boundaries; then at elevated temperatures (1000 °C) the diffusivities of W and Cr are such that the Gibbs energy of W_3_Cr is overcome and the intermetallic is formed. The W-rich equiaxed grains suggest that the initial metastable state of the nano-multilayered structure shifts the kinetics of nucleation to W-rich phases, and in turn these stabilized W-rich solutions have lower Gibbs free energy [50]. In summary, interface coherency affects the GB and interface mobility, but GBE determines the state where the microstructure is stable.

**Figure 5:**
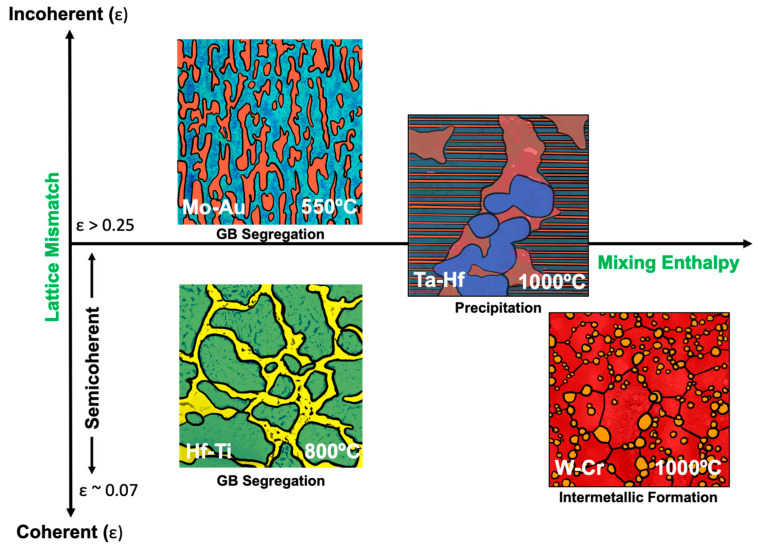
Schematics at selected temperatures highlighting various segregation behaviors across the material systems, noting segregation (Mo-Au, Hf-Ti), precipitation (Ta-Hf), and intermetallic formation (W-Cr) The axes show the magnitude of lattice mismatch and the magnitude of the mixing enthalpy for the binary alloys. Coherency thresholds are indicated as a function of lattice mismatch (ε): incoherent (ε > 0.25). semicoherent (0.07 < ε < 0.25) and coherent (ε < 0.07).

To further visualize the complexity of coherency across the systems, Figure 5 provides a schematic snapshot of various experimentally observed segregation behaviors across the four systems. The previous discussion demonstrated that the initial attributes of the as-sputtered NMM systems (GBE and coherency) influence the final transformed microstructure. During these transformations, many processes are occurring, as indicated by multiple stages of the DSC scans. Various transitions are illustrated, including segregation (Mo-Au, Hf-Ti), precipitation (Ta-Hf) and intermetallic transformation (W-Cr). Furthermore, the systems are organized along the x-axis by their magnitude of mixing enthalpy for the systems and along the y-axis for lattice mismatch and approximate interface coherency. This overview highlights the interplay between system properties—where grain boundary segregation behavior results in drastically different morphologies in the Hf-Ti and Mo-Au; two systems that have similar enthalpies of mixing but markedly different coherency in the initial multilayer structure. Incoherent interfaces in Mo-Au are more mobile and the system exhibits competing mechanisms of GB segregation and phase separation, resulting in segregation zones, as compared to the traditional GB segregation shown in Hf-Ti. In the case of the Ta-Hf and W-Cr multilayers, the possibility of further decreasing the grain boundary energy should have driven a greater intergranular solute enrichment. However, Figure 5 depicts that the bimodal coherency in Ta-Hf yields intricate precipitation behavior due to the competing semicoherent columnar grain and incoherent brick-like grain mobilities. Grain boundary segregation was observed for the Ta-Hf system in the form of a Ta wetting layer around the recrystallized grains at the bottom of the film, where recrystallization was facilitated by the presence of incoherent interfaces at the bottom of the film. In W-Cr, GB complexions were not observed, rather the W_3_Cr intermetallic formed at triple and quadrupole points via continuous precipitation, a process which requires the depletion of Cr to nucleate these intermetallic grains. This behavior is in part due to the high mixing enthalpy of the W-Cr system as well as the higher density of interfaces characteristic of the NMM structure, all of which shift the kinetics of the phase transformation to form W_3_Cr intermetallics and in turn allows for the development of diffusion zones where intermetallics can nucleate.

The four systems explored in this work, varied in terms of composition, coherency, lattice mismatch, as well as alternating layer structure (bcc/fcc, bcc/bcc, hcp/fcc, and bcc/hcp). The systems are unified by the fact that they are metallic binary NMM systems with non-equiatomic alloy ratios, meaning they have a majority solvent element, and all have nanoscale layer thicknesses. This is an important distinction because many NMM studies focus on equiatomic, or repeated layer thickness, systems [22,23,26,28,51,52,53,54,55,56,57,58,59,60] or are concerned with the evolution of microlaminates, where layer thicknesses are >100 nm [61,62]. It has been shown that an unequal alloying content affects transformation behavior [63] and nanoscale layer thicknesses affect the diffusion pathways necessary for transformation [64]. Thus far in this manuscript, we have stressed the importance of understanding the links between initial characteristics (coherency, local and global composition, lattice mismatch, initial grain size, and material selection) and the final transformed microstructure. Although the mechanisms are intricate on the path to a tailored microstructure, key parameters can be distilled: the propensity for a nanometallic multilayer system to transform into a fully equiaxed microstructure can be simplified down to its drivers for transformation. The drivers are dependent on these initial properties that either facilitate or hinder these transformations. Some of these fundamental drivers can be identified as the lattice mismatch, alloy composition, and surface energy, all of which depend on the initial material selection.

**Figure 6:**
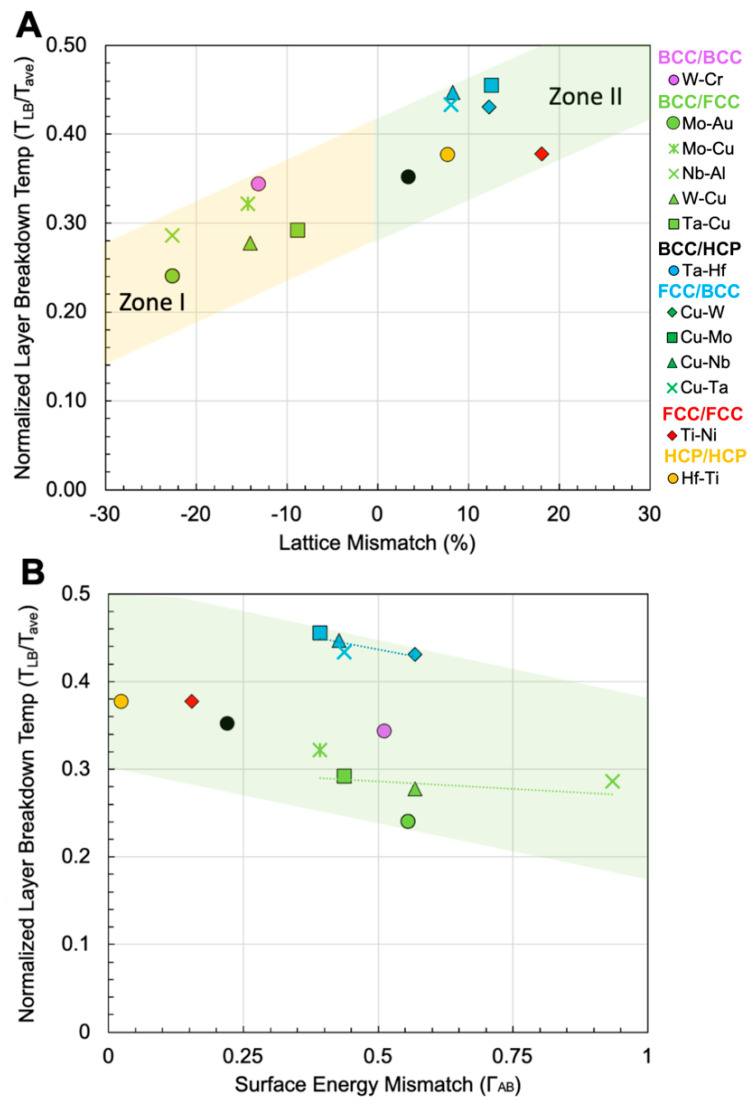
(**A**) Plot of the normalized layer breakdown temperature T_LB_/T_Mave_ (experimentally observed transition to a fully equiaxed microstructure normalized to average melting temperature of binary alloy) as a function of lattice mismatch between the constituent elements for NMM systems. Zone I corresponds to a larger solute lattice spacing than the solvent. Zone II indicates a smaller solute lattice spacing than the solvent. (**B**) Plot of T_LB_/T_Mave_ as a function of surface energy mismatch $\left(\Gamma_{AB}\right)$. Colors of the data points correspond to NMM alternating structure (e.g., bcc/fcc, fcc/bcc, etc.). from this study and other studies [17,18,19,20,52,63,65,66,67,68,69].

To visualize experimental observations over many binary NMM systems, the simplified contributions of geometric and energetic drivers are illustrated in Figure 6. In these plots, NMM structures from the present study and from literature across many material systems [17,18,19,20,52,63,65,66,67,68,69] are presented and are tabulated in Supplementary Table S1. All systems shown are binary, non-equiatomic (>60 at.% base element) nano-multilayered films subjected to heat treatments. It is important to note that the microstructures of these films are predominantly characterized ex situ, and therefore temperatures of transformations are approximate. The systems from this work (W-Cr, Mo-Au, Ta-Hf, Hf-Ti) are denoted by a circle, and the color of each data point indicates the type of alternating layer structure (bcc/bcc, bcc/fcc, etc.) that the NMM comprises. To the best of the authors’ knowledge, this presents all available experimental demonstrations of nano-multilayer to equiaxed microstructural transformations under annealing where the original NMM structure is a non-equiatomic binary system.

In Figure 6A, the lattice mismatch between the solute and solvent elements illustrates a simplified relationship between interface coherency and its effect on microstructural evolution. A negative lattice mismatch (Zone 1) corresponds to a larger solute lattice constant than the solvent, and a positive lattice mismatch (Zone II) corresponds to a smaller solute lattice constant than the solvent. In NMM systems, the transformation to a fully equiaxed microstructure can be marked by the loss of the initial layered structure. Therefore, the layer break-down temperature (T_LB_) describes the temperature at which this transition is experimentally observed. T_LB_ was normalized to the weighted average of the melting temperature (T_Mave_) of the solute and solvent elements of each respective system. The weighted average of the melting temperature can be defined as $T_{Mave}=T_{M}{}_{a}x_{a}+T_{M}{}_{b}x_{b}$, where $T_{M}$ and x are the melting temperatures and global alloy content of each element (a,b). In this plot, higher y-axis positions indicate a higher normalized layer breakdown temperature needed to transform the NMM structure. Therefore, Zone I systems transform into nanocrystalline microstructures more readily with increasing lattice mismatch than Zone II. This plot also suggests that higher lattice mismatch drives a higher normalized layer breakdown temperature. This observation may contradict conventional findings in the literature [40,70,71], where incoherent interfaces should be more mobile, and as a result, require lower temperatures to evolve, regardless of the positive or negative direction of mismatch. Therefore, although lattice mismatch is an important factor, it alone is not sufficient to predict the propensity of a NMM system to transform into an equiaxed structure. Furthermore, the coherency of a system can be described in more detail by using both geometric terms (lattice mismatch, atomic radii ratios) and energetic terms. An additional approach to describe the coherency of a system is by using the surface energy mismatch between the two metals. The structure of interphases can be described as a result of the surface energy (γ) and atomic radius (r) of the individual metals (A,B). If the surface energy mismatch of two metals ($\Gamma_{AB}=2\left(\gamma_{A}-\gamma_{B}\right)/\left(\gamma_{A}+\gamma_{B}\right)$ is $\Gamma_{AB}<0.5$ and the ratio of atomic radii ($r=r_{A}/r_{B}$) is r≤1, then a relatively stable coherent (5–200 mJ·m^2^) or semicoherent (200–800 mJ·m^2^) interphase is formed [41,42]. For example, the Hf-Ti system ($\Gamma_{Hf,Ti}=0.02,\;r_{Hf,\;Ti}=1.53)$ readily forms semicoherent interfaces, evident in the uninterrupted columnar structures with subgrains that persist through as-sputtered layer interfaces. Accordingly, increasing surface energy mismatch between elements A and B in a NMM system should drive microstructural transformations at lower normalized annealing temperatures. This phenomenon is illustrated in Figure 6B, which presents the experimentally observed average layer breakdown temperatures as a function of surface energy mismatch $(\Gamma_{AB}).$ As interfacial surface energy mismatch increases, the temperature (normalized to average melting temperature) required to induce equiaxed microstructures decreases. Therefore, less energetically stable incoherent interfaces have higher driving forces for transformation. Figure 6B uses the same colors of each data point to attribute the datum to a specific NMM structure (bcc/fcc, fcc/fcc, hcp/hcp, etc.) from the present study as well as the other previously identified studies from literature [17,18,19,20,52,63,65,66,67,68,69]. For a given subset of NMM structures (e.g., fcc/bcc), a more pronounced trend of decreasing normalized layer breakdown temperature with increasing surface energy mismatch is evident. Furthermore, for systems with a majority (>60 at.%) fcc structure, the onset of thermal transformations is delayed to higher temperatures as compared to a majority bcc structure. More non-equiatomic NMM systems with varying structures (bcc/hcp, hcp/fcc) should be examined to assess the susceptibility of NMM-to-nanocrystalline transformations as a function of interfacial energy properties. Additionally, in situ annealing studies could help understand and refine the relationships between initial NMM properties and the exact temperatures at which transformations occur [72,73,74].

## 4. Conclusions

In this work, we explored binary NMMs as a means to achieve equiaxed nanostructured materials. Four distinct systems (Mo-Au, Hf-Ti, Ta-Hf, and W-Cr) were examined to provide an overarching study on thoroughly characterized binary films before and after microstructural transformation. The contributions of a variety of initial NMM properties were investigated and the global context for their evolution under thermal annealing was presented. This study demonstrates that both global and local composition, interface energy, and material selection are crucial factors in developing highly tuned nanostructures from initial nano-multilayer designs. In doing so, this work presents a novel experimental overview of well-characterized NMM-to-nanocrystalline transformations across many non-equiatomic material systems where thermal stability has been predicted but not yet experimentally demonstrated. Further studies examining compositional context by comparing non-equiatomic NMM systems to equal layers would be beneficial to fully understand the impact of composition. Additionally, isolating length scale effects (layer thickness, initial grain size) on the pathway from layered to equiaxed structures would be a valuable area of study. Overall, this work serves as a guide on how to achieve tailored nanoscale materials through careful selection of initial characteristics in binary NMMs.

## Figures and Tables

**Table 1:** Summary of as-sputtered multilayer film characteristics.

Alloy System	Global Composition	Alloy Layer Composition	Layer Thicknesses	Total Film Thickness (µm)	Layers
Hf-Ti	20.1 at.% Ti	9.7 at.% Ti	40 bilayers: 5.3 nm Ti/48.1 nm Hf-Ti	2.1	hcp/hcp
Ta-Hf	22.4 at.% Hf	14.6 at.% Hf	127 bilayers: 1.7 nm Hf/14.0 nm Ta-Hf	2.0	bcc/hcp
W-Cr	33.1 at.% Cr	12.4 at.% Cr	247 bilayers: 1.5 nm Cr/6.3 nm W-Cr	2.0	bcc/bcc
Mo-Au	21.3 at.% Au	11.0 at.% Au	107 bilayers: 2.5 nm Au/17.3 nm Mo-Au	2.0	bcc/fcc

## Data Availability

The data in this study is available upon request.

## Supplementary Materials

The following supporting information can be downloaded at: https://www.mdpi.com/article/10.3390/ma15010382/s1, Table S1: Supplementary data for Figure 6 summarizing geometry parameters, surface energy parameters, and experimentally observed layer breakdown temperatures. All referenced data sets are non-equiatomic binary NMM structures with experimentally observed microstructural transformations under heat treatment.
