# Supplementary material for: An Overview of Nano Multilayers as Model Systems for Developing Nanoscale Microstructures
Source: Materials (Basel). 2022 Jan 5;15(1):382. doi: 10.3390/ma15010382 (PMC8746540; doi:10.3390/ma15010382)
Supplement: Supplementary file 1 [file materials-15-00382-s001.zip › materials-1489597-supplementary.pdf]

materials MDPI

Supplementary Materials: An Overview of Nano Multilayers as Model Systems for Developing Nanoscale Microstructures

Chelsea D. Appleget [1], Juan Sebastian Riano [2] and Andrea M. Hodge [1,2,*]

[1] Department of Aerospace and Mechanical Engineering, University of Southern California, Los Angeles, CA 90089, USA; capplege@usc.edu
[2] Mork Family Department of Chemical Engineering and Materials Science, University of Southern California, Los Angeles, CA 90089, USA; rianozam@usc.edu
* Correspondence: ahodge@usc.edu; Tel.: +1-213-740-4225

Table S1. Supplementary data for Figure 6 summarizing geometry parameters, surface energy parameters, and experimentally observed layer breakdown temperatures. All referenced data sets are nonequiatomic binary NMM structures with experimentally observed microstructural transformations under heat treatment.

System	Alternating Layer Structure	Lattice Mismatch (%)	Geometry Parameter (r = b/a)	Surface Energy Mismatch	Layer Breakdown Temp (°C)	Norm. Average layer Breakdown Temp
Mo-Au [1]	BCC/FCC	−22.58	0.95	0.56	550	0.24
W-Cr [2]	BCC/BCC	−13.15	1.10	0.51	1000	0.34
Hf-Ti [3]	HCP/FCC	7.68	1.53	0.02	800	0.38
Ta-Hf [4]	BCC/HCP	3.40	0.96	0.22	1000	0.35
Ti-Ni [5]	FCC/FCC	18.10	1.18	0.15	600	0.38
W-Cu [6]	BCC/FCC	−14.01	1.07	0.57	800	0.28
Cu-W [6]	FCC/BCC	12.29	0.93	0.57	700	0.43
Nb-Al [7]	BCC/FCC	−22.59	0.78	0.93	600	0.29
Mo-Cu [8]	BCC/FCC	−14.30	1.07	0.39	800	0.32
Cu-Mo [8]	FCC/BCC	12.51	0.94	0.39	725	0.46
Cu-Nb [9]	FCC/BCC	8.25	0.89	0.43	607	0.45
Ta-Cu [10]	BCC/FCC	−8.81	1.12	0.44	700	0.29
Cu-Ta [11]	FCC/BCC	8.10	0.89	0.44	800	0.43

References

1. Bahena, J.A.; Riano, J.S.; Chellali, M.R.; Boll, T.; Hodge, A.M. Thermally activated microstructural evolution of sputtered nanostructured Mo–Au. *Materialia* **2018**, *4*, 157–165.
2. Riano, J.S.; Hodge, A.M. Phase transformations in the W–Cr system at the nanoscale. *Materialia* **2018**, *2*, 190–195.
3. Riano, J.S.; Hodge, A.M. Exploring the microstructural evolution of Hf-Ti: From nanometallic multilayers to nanostructures. *Scr. Mater.* **2018**, *142*, 55–60.
4. Riano, J.S.; Hodge, A.M. Exploring the thermal stability of a bimodal nanoscale multilayered system. *Scr. Mater.* **2019**, *166*, 19–23.
5. Shi, J.; Cao, Z.; Liu, Y.; Zhao, Z. Size dependent alloying and plastic deformation behaviors of Ti/Ni nano-multilayers. *J. Alloys Compd.* **2017**, *727*, 691–695.
6. Druzhinin, A.; Ariosa, D.; Siol, S.; Ott, N.; Straumal, B.; Janczak-Rusch, J.; Jeurgens, L.; Cancellieri, C. Effect of the individual layer thickness on the transformation of Cu/W nano-multilayers into nanocomposites. *Materialia* **2019**, *7*, 100400.
7. Im, Y.; Johnson, P.; McKnelly Jr, L.; Morris Jr, J. Nb3Al formation in sputter-deposited Nb/Al multilayer samples. *Journal of the Less Common Metals* **1988**, *139*, 87–95.
8. Srinivasan, D.; Sanyal, S.; Corderman, R.; Subramanian, P. Thermally stable nanomultilayer films of Cu/Mo. *Metall. Mater. Trans. A* **2006**, *37*, 995–1003.
9. Troche, P.; Hoffmann, J.; Heinemann, K.; Hartung, F.; Schmitz, G.; Freyhardt, H.C.; Rudolph, D.; Thieme, J.; Guttmann, P. Thermally driven shape instabilities of Nb/Cu multilayer structures: Instability of Nb/Cu multilayers. *Thin Solid Films* **1999**, *353*, 33–39.
10. Zhang, Y.; Fu, L.; Zhu, J.; Yang, W.; Li, D.; Zhou, L. Thermal stability of co-sputtered TaCu amorphous nano-multilayers. *Thin Solid Films* **2021**, 138766.
11. Lee, H.-J.; Kwon, K.-W.; Ryu, C.; Sinclair, R. Thermal stability of a Cu/Ta multilayer: An intriguing interfacial reaction. *Acta. Mater.* **1999**, *47*, 3965–3975.
